# Epidemiology of traumatic brain injury in Europe

**DOI:** 10.1007/s00701-015-2512-7

**Published:** 2015-08-14

**Authors:** Wouter Peeters, Ruben van den Brande, Suzanne Polinder, Alexandra Brazinova, Ewout W. Steyerberg, Hester F. Lingsma, Andrew I. R. Maas

**Affiliations:** University of Antwerp, Antwerp, Belgium; Department of Public Health, Centre for Medical Decision Making, Erasmus MC, Rotterdam, The Netherlands; Faculty of Health Sciences and Social Work, Trnava University, Trnava, Slovak Republic; Department of Neurosurgery, Antwerp University Hospital, Wilrijkstraat 10, 2650 Edegem, Belgium

**Keywords:** Epidemiology, Traumatic brain injury, Systematic review, Incidence, External cause, Mortality

## Abstract

**Background:**

Traumatic brain injury (TBI) is a critical public health and socio-economic problem throughout the world, making epidemiological monitoring of incidence, prevalence and outcome of TBI necessary. We aimed to describe the epidemiology of traumatic brain injury in Europe and to evaluate the methodology of incidence studies.

**Method:**

We performed a systematic review and meta-analyses of articles describing the epidemiology of TBI in European countries. A search was conducted in the PubMed electronic database using the terms: epidemiology, incidence, brain injur*, head injur* and Europe. Only articles published in English and reporting on data collected in Europe between 1990 and 2014 were included.

**Results:**

In total, 28 epidemiological studies on TBI from 16 European countries were identified in the literature. A great variation was found in case definitions and case ascertainment between studies. Falls and road traffic accidents (RTA) were the two most frequent causes of TBI, with falls being reported more frequently than RTA. In most of the studies a peak TBI incidence was seen in the oldest age groups. In the meta-analysis, an overall incidence rate of 262 per 100,000 for admitted TBI was derived.

**Conclusions:**

Interpretation of published epidemiologic studies is confounded by differences in inclusion criteria and case ascertainment. Nevertheless, changes in epidemiological patterns are found: falls are now the most common cause of TBI, most notably in elderly patients. Improvement of the quality of standardised data collection for TBI is mandatory for reliable monitoring of epidemiological trends and to inform appropriate targeting of prevention campaigns.

**Electronic supplementary material:**

The online version of this article (doi:10.1007/s00701-015-2512-7) contains supplementary material, which is available to authorised users.

## Introduction

Traumatic brain injury (TBI) constitutes a major health and socioeconomic problem throughout the world [6, 9]. It is prevalent in both low- and high-income countries and affects people of all ages. TBI is called the ‘silent epidemic’ because problems resulting from TBI are often not immediately visible, and TBI patients are not very vociferous. The term ‘silent’ further reflects the common underestimation of the actual incidence and that society is often unaware of the impact of TBI [14]. Epidemiological studies of TBI are essential to the targeted prevention and effective treatment of brain-injured patients.

Epidemiological studies are, however, often confounded by a general lack of clear definitions for TBI. A clear, concise definition of TBI is essential in the attempt to understand the epidemiology.

‘Traumatic brain injury’ has replaced the former term ‘head injury’ as it better captures the importance of the ‘brain’ [28]. TBI was recently defined as: ‘An alteration in brain function, or other evidence of brain pathology, caused by an external force’ [23].

Tagliaferri et al. [38] conducted a systematic review on the epidemiology of TBI in Europe in 2006. In their review they analysed 23 studies published between 1980 and 2003. An aggregated (i.e. fatal plus hospitalised) incidence rate of 235 cases per 100,000 people per year, an average mortality rate of 15 per 100,000 people per year and a case fatality rate of 2.7 % were calculated.

In the past decade, new insights into the epidemiology of TBI have emerged. Epidemiological patterns appear to be changing with an increasing incidence of TBI in the elderly. Various reports claim that mortality in TBI is decreasing [8, 15]. The purpose of this systematic review is to provide a contemporary overview of epidemiology of TBI in Europe with a specific focus on epidemiological patterns and on the methodological quality of epidemiologic studies.

## Methods

A search was conducted in the PubMed electronic database using the following search-terms: epidemiology, incidence, brain injur*, head injur* and Europe. Reference lists of review studies and articles included in the review were screened for titles that included the key terms.

### Inclusion criteria

Studies were included if they met the following inclusion criteria: (1) published in English in the period 1990–2014 with a full text available; (2) original study; (3) predominantly focusing on the epidemiology of TBI; (4) predominantly focusing on TBI, not on the more general head injury; (5) focusing on the population as a whole, not only on a specific subgroup (e.g. cyclists, rugby players, children, etc.); (6) study period at least 1 year; (7) only including data from 1990 or later; (8) not only focusing on mild TBI; (9) if multiple publications used the same study population, the most recent report was used, as it generally addressed a larger population.

### Data extraction

Relevant papers were selected by screening the titles (first step), abstracts (second step) and entire articles (third step), retrieved through the database searches. During each step the title, abstract or entire article was screened to ensure that it met the inclusion criteria. This screening was conducted independently by two researchers (W.P. and R.v.d.B.). Extracted data included source population, study period, study group size, case ascertainment, case criteria, incidence, age distribution, sex distribution, mortality and most frequent cause of TBI.

### Methodological quality

Characteristics and methodological quality of selected studies were evaluated with a particular focus on study design, case ascertainment, case definition, patient population and the description of the methodology. We based the evaluation of methodological quality on five elements of the STROBE checklist [39] which were most relevant to the quality of reported incidence and mortality rates: study design, setting, participants, data sources/measurement and study size.

### Data and statistical analysis

Data are reported as in the original manuscripts. For calculation of an overall incidence rate in the meta-analysis, we used random effects modelling to address heterogeneity between the studies. Heterogeneity was expressed by the τ^2^ and *I*^2^ statistics. Tau-squared represents the estimate of the between-study variants in a random effects meta-analysis. A τ^2^ > 1 suggests the presence of substantial statistical heterogeneity. *I*^2^ represents the percentage of the total variation across studies due to heterogeneity [11]. Comprehensive Meta-Analysis (CMA) software was used for the calculations.

## Results

**Fig. 1:**
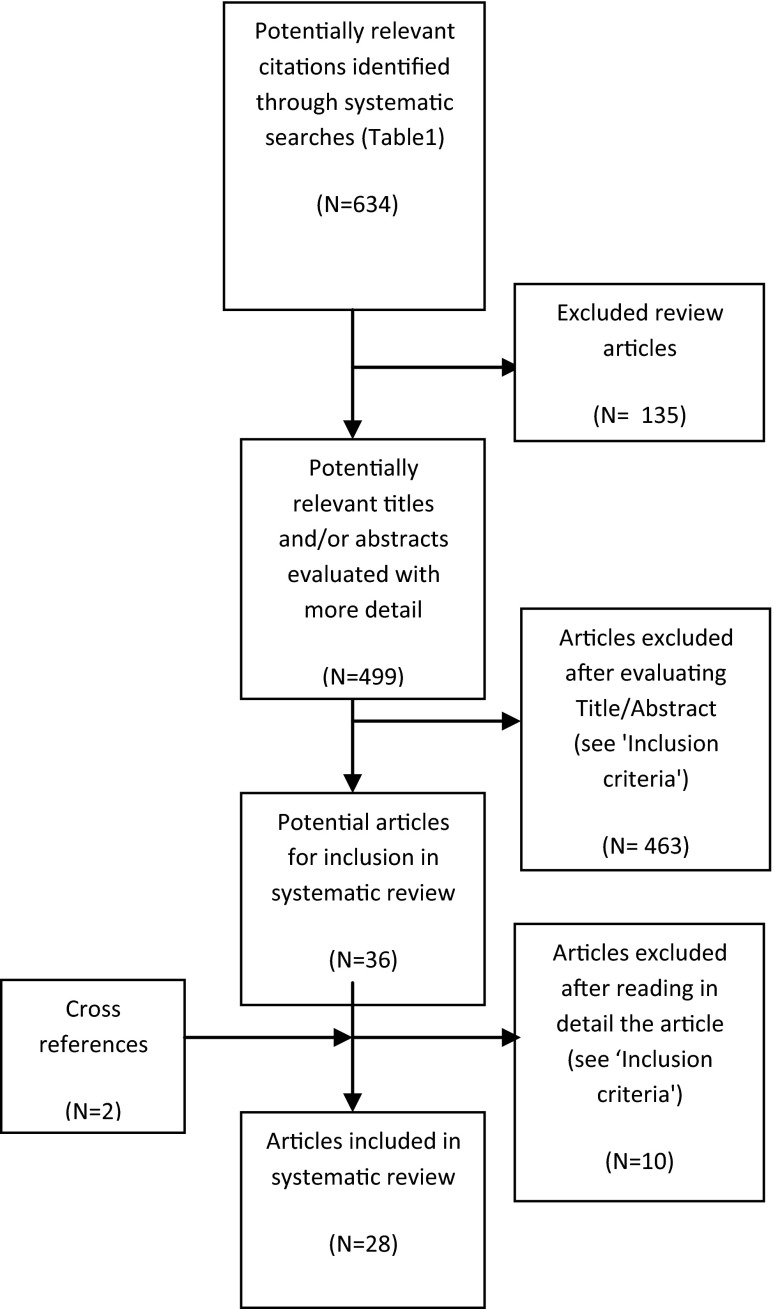
Flow diagram of the literature search and selection of articles

The PubMed search identified 743 articles; 109 duplicates were removed, resulting in 634 potentially relevant citations (*see* ESM 1). Following the screening of titles, abstracts and entire articles, a total of 28 articles were retained for inclusion in this systematic review (Fig. 1).

### Study characteristics

Eight reports were of national populations (Austria, Finland, Germany [2×], Norway, Scotland, Spain and Netherlands). One study compared the epidemiology of TBI between regions of different European countries [22]. Nineteen focused on regions, counties or provinces of one European country. Altogether we found data from sixteen different countries: Norway, Sweden, Netherlands, Italy, Germany, Greece, Finland, France, Austria, Slovak Republic, Croatia, Macedonia, Bosnia, Poland and Scotland.

Fifteen out of the 28 studies had a study period of exactly 1 year, five studies [14, 18, 25, 33, 35] had a study period of 10 years or more. The number of included patients ranged from 247 [12] to 280,000 [7], the size of the total source population from 83,900 [24] to 82,037,100 [34]. Nine studies did not report their source population size. Characteristics of the included studies and results of quality assessment are presented in Table 1.

**Table 1:** Study characteristics and quality assessment

Reference	Source Population	Study duration and period	Study group size (included patients/source population)	5 STROBE quality criteria
Andelic et al. (2008) [2]	Population of city Oslo, Norway	1 year (15 May 2005–14 May 2006)	445/543,129	Complete
Andelic et al. (2012) [1]	Population of Norway	2 years (2009–2010)	359/2,143,661	Complete
Andersson et al. (2003) [3]	Population based data from region of Western Sweden	1 year (April 1992-April 1993)	753/138,000	Complete
Andriessen et al. (2011) [4]	Case series: adults (>16 years) admitted to one of the 5 participating specialised trauma centres in The Netherlands	1 year (June 2008-May 2009)	508/not reported	Incomplete: participants
Baldo et al. (2003) [5]	Residents of the Veneto Region of Northeast Italy	5 years (1996–2000)	55,368/4,480,000	Complete
Firsching & Woischneck (2001) [7]	Population of Germany	1 year (1996)	280,000/82,000,000	Incomplete: study design, participants
Heskestad et al. (2009) [10]	Residents of the Stavanger Region, Norway	1 year (2003)	585/283,317	Complete
Ingebritsen et al. (1998) [12]	Population of city of Tromsø, Norway and 16 surrounding municipalities	1 year (1993)	247/108,017	Complete
Katsaragakis et al. (2010) [13]	Case series: patients of 30 hospitals in Greece. Hospitals not reported.	1 year (year not reported)	3,383/not reported	Incomplete: setting, participants, data sources/measurement
Koskinen & Alaranta (2008) [14]	All residents of Finland	15 years (1991–2005)	77,959/5,010,000 (1991)-5,250,000 (2005)	Complete
Maegele et al. (2007) [18]	Residents of the Cologne area, Germany	10 years (1990–1999)	731/1,000,000	Complete
Masson et al. (2001) [19]	Population of Aquitaine, France	1 year (1996)	325/2,800,000	Complete
Masson et al. (2003) [20]	Population of Aquitaine, France	1 year (1996)	497/2,800,000	Complete
Mauritz et al. (2008) [22]	European Regions with different economic status (Austria [‘high income’], Solvakia and Croatia [‘upper middle income’], Macedonia and Bosnia [‘lower middle income’])	4.5 years (January 2001-June 2005)	1,172/not reported	Complete
Mauritz et al. (2014) [21]	All Austrian residents	3 years (2009–2011)	74,744/8,443,018	Complete
Numminen (2010) [24]	Population based data from region of South East Finland	2 years (April 2002- March 2004)	370/83,900	Complete
Pérez et al. (2012) [25]	Residents of Spain, 2000-2009	10 years (2000–2009)	206,503/not reported	Incomplete: study size
Puljula et al. (2013) [26]	All residents of Northern Ostrobothnia, Finland, 1999 and 2007	2 years (1999 & 2007)	126 (1999)- 135 (2007)/ 369,827 (1999)- 390,038 (2007)	Complete
Rickels et al. (2010) [27]	Residents in regions of Hannover and Münster, Germany	1 year (March 2000-Februari 2001)	6,783/2,200,000	Complete
Rosso et al. (2007) [29]	Case series: patients from five centres (Graz, Klagenfurt, Linz, Salzburg, Vienna) in Austria	3 years (between 1999 and 2004)	492/not reported	Complete
Scholten et al. (2014) [30]	Population of The Netherlands	3 years (2010–2012)	3,762/not reported (study); 34,681/not reported (national estimate)	Incomplete: setting, study size
Servadei et al. (2002) [31]	Residents of the Romagna Region of Italy	1 year (1996)	2,430/970,000	Complete
Servadei et al. (2002) [32]	Residents in regions of Trentino and Romagna, Italy	1 year (1998)	1,562 (Trentino)-2,880 (Romagna)/470,000 (Trentino)-970,000 (Romagna)	Complete
Shivaji et al. (2014) [33]	Population of Scotland	12 years (1998–2009)	208,195/not reported	Incomplete: study size
Steudel et al. (2005) [34]	Total German population	1 year (1998)	276,584/82,037,100	Complete
Stocchetti et al. (2012) [35]	Patients admitted to three neurosurgical ICUs in Milan and Monza, Italy, 1997-2007	11 years (January 1997-December 2007)	1,366/not reported	Incomplete: participants
Styrke et al. (2007) [36]	Population-based data from region of Northern Sweden	1 year (2001)	449/137,000	Incomplete: setting
Szarpak & Madziala (2011) [37]	Residents of the Piaseczno and Otwock Counties, Poland	1 year (2009)	1,049/not reported	Incomplete: setting, participants, study size

### Methodological quality and incidence

A total of 19 studies met the five selected STROBE criteria. Nine studies did not meet all five criteria, of which two failed on two criteria and a further 2 on three criteria (Table 1). Table 2 summarises details of inclusion criteria, case definitions, severity assessment and reported/calculated incidence rates per year of the selected studies. A large variation was found in inclusion criteria, case ascertainment and case definitions. Eight studies were based on hospital admissions, six on emergency department admissions and four on a combination of both. Other sources used for case ascertainment were death certificates, ICU admissions, hospital discharges, pre-hospital emergencies, or a combination of these. We also found large differences in the case criteria that were used in the studies. Seven studies used ICD-10 codes to define TBI, seven used ICD-9 codes and another two used both. Five studies used the GCS. Other tools that were used to define TBI, were Head Injury Severity Scale (HISS), Abbreviated Injury Scale (AIS) or clinical symptoms. Twenty-one out of 28 studies provided information on the severity distribution of TBI. The severity of TBI was measured by the GCS score in 12 out of these 21 studies. Other methods that have been used to measure the TBI severity were AIS head score, HISS score, or ICD codes. Eight out of 21 studies focused on severe or moderate-to-severe TBIs. In studies that provide complete information on all TBI severities (*n* = 12; [2, 3, 5, 7, 10, 12, 24, 27, 31, 34, 36, 37]), we see that the percentage of mild TBIs varies between 71 % [24] and 97.5 % [3].

**Table 2:** Inclusion criteria and incidence rate

Reference	Inclusion criteria and case ascertainment	Case definitions	TBI severity	Incidence rate/year
Andelic et al. (2008) [2]	Persons residing in Oslo at the time of injury, hospitalised with acute TBI, during the period 2005–2006.	ICD-10 codes: S02.0-S02.9, S06.0-S06.9, S07.0, S07.1, S07.8, S07.9, S09.7-S09.9, T04 and T06. Excluded: isolated injuries to scalp, isolated facial and jaw fractures, anoxia, birth trauma, patients not living in Oslo, patients with subdural haematomas, with multiple admissions for same injury and patients admitted later than 48 h after the trauma.	GCS: 86 % mild, 7.9 % moderate, 6.1 % severe	83.3/105
Andelic et al. (2012) [1]	All adults (>16 years old) residing in Norway with severe TBI admitted within 72 h after injury to a Norwegian Trauma Referral Centres during the 2-year period.	ICD-10 codes S06.0-S06.9. Severe TBI was defined as lowest unsedated GCS Score ≤8 during the first 24 h after injury.	GCS: 100 % severe	5.2/ 105 (2009) 4.1/105 (2010) (Overall age-adjusted incidence rate)
Andersson et al. (2003) [3]	Patients attending hospital emergency unit, discharge register, regional neurosurgical clinic and coroner’s records.	ICD-9 codes 850–854, 800–804 plus mix of clinical symptoms or signs as defined by American Congress of Rehabilitation Medicine for TBI severity.	ACRM criteria: 97.5 % mild, 2.5 % moderate-to-severe	546/105
Andriessen et al. (2011) [4]	Patients with TBI admitted to emergency department of one of the trauma centres.	Patients with TBI and an ED admission GCS score ≤13. TBI not further defined. Exclusion criteria: age <16 years and hospital admission >72 h after injury.	GCS: 34 % moderate, 67 % severe	Not reported
Baldo et al. (2003) [5]	All hospital discharge records containing ICD-9-CM codes: 800.0–801.9, 803.0–804.9, 850.0–854.1.	Brain injury defined by discharge ICD codes and only cases hospitalised.	ICD/AIS: 1996 = 45 % mild, 14 % moderate, 6 % severe, 35 % unknown; 1998 = 43 % mild, 16 % moderate, 7 % severe, 33 % unknown; 2000 = 53 % mild, 13 % moderate, 18 % severe, 16 % unknown	301/105 (1996) 249/105 (1998) 212/105 (2000) (29.4 % decrease from 1996 to 2000)
Firsching & Woischneck (2001) [7]	Data from death certificates, Federal Board of Statistics (Hospital discharge reports).	ICD-9 codes (not reported) for hospital admitted persons.	73 % mild	350/ 105 (overall) 247/ 105 (mild) with intracranial lesions = 29/105 with skull fractures = 21/105
Heskestad et al. (2009) [10]	All head-injured patients (*n* = 585) referred to any department at the University Hospital of Stavanger during a 1-year period (2003).	ICD-10 codes S00 through S09 with subgroups. Head injury was defined as physical damage to the brain or skull caused by external force. Isolated injuries to the scalp, face or cervical spine and patients with birth injuries were excluded.	HISS: 26 % minimal, 58 % mild, 3 % moderate, 13 % severe	207/105 (overall) hospital admission rate of 157/105
Ingebritsen et al. (1998) [12]	All head-injured patients referred first to University Hospital or admitted to any hospital department plus emergency department treated and discharged.	Head injury defined as physical damage to the brain or skull by external force and GCS and Head Injury Severity Scale.	HISS: 32 % minimal, 49 % mild	229/105 (overall) hospital admission rate of 169/105
Katsaragakis et al. (2010) [13]	Trauma patients that required admission, transfer to a higher level unit or arrived dead or died in the emergency department and had had at least one brain injury.	Brain injury not defined.	Not reported	Not reported
Koskinen & Alaranta (2008) [14]	Hospital Discharge register of the Finnish National Research Development Centre for Welfare and Health for entire 5.1 million population.	ICD-9 codes 800-801, 803, 850-854, first time admissions during 1991-96. ICD-10 codes S02.0, S02.00, S02.01, S02.1, S02.10, S02.11, S02.7, S02.70, S02.71, S02.8, S02.80, S02.81, S02.9, S02.90, S02.91, T020, S06.0, S06.1-9, first time admissions during 1997-2005.	Not reported	97/105 (1991-1995) 102/105 (1996-2000) 104/105 (2001-2005)
Maegele et al. (2007) [18]	130,000 pre-hospital emergencies were screened for TBI.	Patients with a pre-hospital GCS score ≤8 and/or AIS head score ≥2 with confirmed TBI via appropriate diagnostic tests (e.g. CT).	GCS/AIS head: 100 % severe	7.3/105
Masson et al. (2001) [19]	Persons admitted to hospital via an emergency service with diagnosis of severe brain injury during 1996.	Severe brain injury defined by AIS score of 4 or 5 to head region.	GCS: 100 % severe	17.3/105 (overall) 7.2/105 (AIS head 4) 10.1/105 (AIS head 5)
Masson et al. (2003) [20]	Patients admitted to any one of 19 public hospitals with prolonged coma.	Persons with prolonged coma or significant intra-cranial injury with coma > 24 h: coma determined from GCS of 8 or less before sedation.	AIS head: 100 % severe	8.5/105 (248 patients registered)
Mauritz et al. (2008) [22]	Patients with severe TBI admitted to one of the 13 tertiary-care-level centres.	Severe TBI according to the criteria defined by the US National Traumatic Coma Database: GCS score ≤8 following resuscitation or a GCS score deteriorating to ≤8 within 48 h of injury.	GCS: 100 % severe	Not reported
Mauritz et al. (2014) [21]	Data on all hospital discharges, outpatients and in-hospital deaths due to TBI were collected from various sources (Statistik Austria, AUVA).	All hospital discharges: ICD-10 codes S06.0–S06.9, T68, or T07; Outpatients: ICD-10 code S06.0–S06.9; In-hospital deaths: ICD-10 codes S01.0–S01.9, S02.0, S02.1, S02.7, S06.0–S06.9, T01.0, T02.0, T04.0, T06.0, T90.1, T90.2, or T90.4–T90.9.	Not reported	303/105
Numminen (2011) [24]	All cases (>14 years) with symptoms of brain injury after head trauma were collected from the health centres in the area covering three municipalities (Imatra, Joutenso and Lappeenranta) and from the one hospital (South Karelia Central Hospital) taking care of all corresponding TBI cases. Also death certificates were collected.	ICD-10 codes S06. Also the death certificates of patients whose main or immediate cause of death was an ICD-10 code of S06 or S07 were included.	GCS/CT: 71 % mild, 29 % severe	221/105 mild TBI in 71 % of patients
Pérez et al. (2012) [25]	National Hospital Discharge Register.	Emergency admissions with ICD-9 codes: 800, 801, 803, 804, 850-854. Programmed and re-admissions were excluded.	ISS: 41.1 % moderate, 26.8 % serious, 32.2 % severe	47.3/105 (during the 9-year study period the incidence rate presents a reduction of 23.8 %)
Puljula et al. (2013) [26]	Patients with moderate-to-severe TBI who were admitted to the ER of Oulo University Hospital, plus those who succumbed from TBI outside the hospital. Only residents of Northern Ostrobothnia were included.	Moderate-to-severe TBI defined as GCS ≤12.	GCS: 100 % moderate-to-severe	34/105 (1999) 35/ 105 (2007)
Rickels et al. (2010) [27]	Patients admitted to hospital emergency department in the regions due to an acute head injury with involvement of the brain.	At least one of the following symptoms or ICD-10 diagnosis codes: Symptoms: nausea or vomiting, headache, loss of consciousness with anterograde/retrograde amnesia, impaired consciousness or impaired vigilance, fracture of face and/or skull, and focal neurological symptom. ICD-10 codes: S02 without S02.5, S04, S06-S07, S09.	GCS: 90.2 % mild, 3.9 % moderate, 5.2 % severe	332/105
Rosso et al. (2007) [29]	Patients admitted to one of the five Austrian hospitals.	Glasgow Coma Scale (GCS) score of 8 or less following resuscitation, which may include endotracheal intubation; or GCS score deteriorating to 8 or less within 48 h of injury.	GCS: 100 % severe	Not reported
Scholten et al. (2014) [30]	All patients with TBI treated at an ED and/or admitted to hospital in The Netherlands in the period 2010-2012. TBI cases were extracted from the Dutch Injury Surveillance System (LIS) and the National Hospital Discharge Registry (LMR). LIS is based upon the registration of 13 hospitals in The Netherlands (12-15 % coverage).	For patients treated at the emergency department, TBI was defined as having a ‘concussion’ or ’other skull-brain injury’ in at least one of the three injuries that can be recorded in LIS. For hospitalised patients, TBI was defined using ICD-9 codes: 850, 800-801, 803, 804, 851-854, 905, 907, 950, 959.	Not reported	213.6/105
Servadei et al. (2002) [31]	Patients admitted to any one of the 7 hospitals in Romagna plus pre- and in-hospital deaths.	ICD-9 codes: 800.0-800.3, 801.0-801.3, 803.0-803.3, 850, 851.0-851.1, 852.0-852.1, 853.0-853.1, 854.0-854.1 with physician diagnosed TBI. Emergency department patients treated and released were excluded.	ICD-9 codes: 81 % mild	250/105 81 % were mild
Servadei et al. (2002) [32]	Medical records of hospital admissions for head injury.	ICD-9 codes: 800.0-800.3, 801.0-801.3, 803.0-803.3, 850, 851.0-851.1, 852.0-852.1, 853.0-853.1, 854.0-854.1.	Not reported	314/105 (overall) 322/105 (Trentino) 297/105 (Romagna)
Shivaji et al. (2014) [33]	Data from Scottish Morbidity Record (SMR01) data-set. SMR01 includes all inpatients and day cases discharged from hospitals across Scotland.	ICD-10 codes: S01.0, S01.9, S02.0, S02.1, S02.3, S02.7, S02.9, S04.0, S06.0, S06.9, S07.0, S07.1, S07.8, S07.9, S09.7, S09.9, T01.0, T02.0, T04.0, T06.0, T90.1, T90.2, T90.4, T90.5, T90.8, T90.9	Not reported	446.4/105 (men) 194.8/105 (women)
Steudel et al. (2005) [34]	Federal Bureau of Statistics, hospital admissions register and mortality register.	ICD 9th = 800-804, 850-854; ICD 10th = S02.0-S02.9 and S06.0-S06.9.	ICD-9 codes: 72 % mild	337/105 (1998)
Stocchetti et al. (2012) [35]	Admission to neurosurgical ICUs.	Admission because of head trauma, with or without extracranial injuries; brain injury severity requiring admission to ICU; time trauma-arrival <24 h; age over 18 years. Brain injury not further defined.	Not reported	Not reported
Styrke et al. (2007) [36]	Data set from the Umeå University Hospital’s injury register.	ICD numbers for brain injuries included are within S06, also the ‘unspecified’ ICD codes were scrutinised to find ‘hidden’ cases.	GCS: 97 % mild, 1 % moderate, 2 % severe	354/105
Szarpak & Madziala (2011) [37]	Based on the emergency intervention cards of Emergency Medical Service teams.	Cranio-cerebral injuries: concussion, open head wound, integument contusion, skull fracture.	GCS: 81 % mild, 10 % moderate, 9 % serious	Not reported

**Fig. 2:**
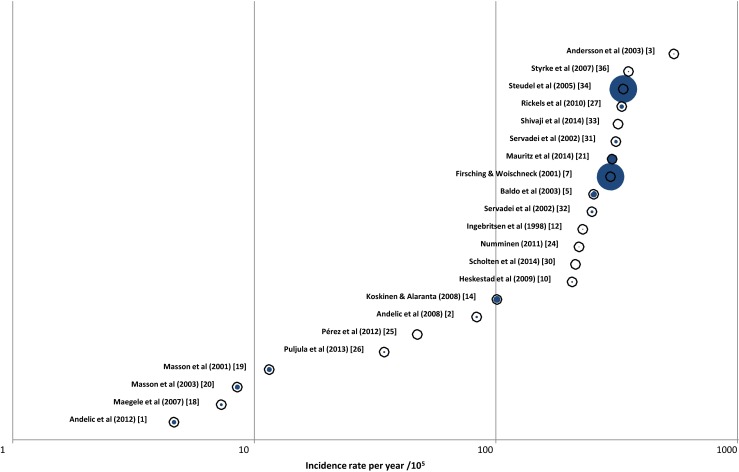
Reported incidence rates for TBI. Rates are expressed per 100,000 population. Each study is marked by an *open circle*; the *size of the blue centre* is proportional to the size of the population under study

These differences make it difficult to compare the incidence. Six out of 28 studies did not report an incidence rate. Out of the remaining 22 studies, five focused on severe or moderate-to-severe TBI [1, 18–20, 26]. The other 17 studies focused on patients with all TBI severities. The incidences of these 17 studies displayed a large variation: Pérez et al. (2011) [25] reported an incidence rate of 47.3 per 10^5^ population per year in Spain in 2000–2009, while Andersson et al. (2003) [3] reported a rate of 546 per 10^5^ population per year in Western Sweden in 1992–1993. Including only the studies that focus on patients with severe TBI (*n* = 4), a range of incidence is reported from 4.1 per 10^5^ population in Norway [1] to 17.3 per 10^5^ population in Aquitaine, France [19]. Fig. 2 illustrates this wide variation of reported incidence rates. We note that studies concentrating on severe TBI [1, 18–20] cluster to the left (low incidence) and those including all injuries to the right (higher incidence).

**Fig. 3:**
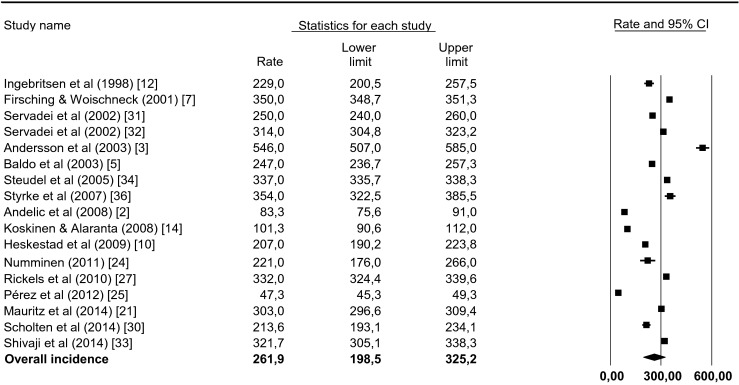
Forest plot of incidence rate per study sorted by year of publication. The forest plot represents the meta-analysis on 17 studies focusing on patients with all TBI severities. A random effects model was applied. Incidence rates are denoted by the *black boxes* and the 95 % CIs by the horizontal lines. The overall incidence rate is represented by the *black diamond*, where the *diamond width* correspondents to the 95 % CIs. Heterogeneity is substantial: τ^2^ = 17650.3; χ^2^ = 72801.5, *df* = 16 (*p* = 0.000); *I* ^2^ = 99.9 %

A meta-analysis of the 17 studies focusing on patients with all TBI severities was performed. Figure 3 shows the large variation of these incidences and a substantial degree of heterogeneity was confirmed on statistical evaluation (*I*^2^ = 99.9 %; *Z* = 6.687). An overall incidence rate of 262 (CI, 185–339) per 100,000 per year for admitted TBI patients was derived.

### Epidemiological patterns: age, sex and cause of TBI

Table 3 presents demographic data of the study populations. In assessing the age distribution, we must note that some studies only include adults in their study population. With this caveat in mind, we see that, in general, TBI is more prevalent among people aged <25 years and among people >75 years. In three studies [14, 18, 26] an increase is seen in the elderly percentile or the mean age over the years of the study.

**Table 3:** Epidemiological patterns: age and sex

Reference	Mean age	Male-to-female ratio
Ingebritsen et al. (1998) [12]	–	1.7:1.0
Masson et al. (2001) [19]	AIS head 4: 44 years	2.5:1.0
	AIS head 5: 45 years	
Firshing & Woischneck (2001) [7]	39 years	2.45:1.0
Servadei et al. (2002) [31]	–	1.6:1.0
Servadei et al. (2002) [32]	–	Romagna: 1.6:1.0
		Trentino: 1.8:1.0
Masson et al. (2003) [20]	41 years	3.1:1.0
Baldo et al. (2003) [5]	Male, 37.7 years	1.55:1.0
	Female, 45.6 years.	
Andersson et al. (2003) [3]	Age, 27 years	1.46:1.0
Steudel et al. (2005) [34]	–	Not reported
Styrke et al. (2007) [36]	Male, 23 years	1.2:1.0
	Female, 22 years	
Rosso et al. (2007) [29]	48.2 years	Overall: 2.6:1.0
		Salzburg: 1.5:1.0
Maegele et al. (2007) [18]	40.3 years	2.7: 1.0
Koskinen & Alaranta (2008) [14]	1.4–1.5:1.0	
Mauritz et al. (2008) [22]	49 years	HI: 2.6:1.0
		UMI: 4.6:1.0
		LMI: 3.5:1.0
Andelic et al. (2008) [2]	29 years. Male, 29 years. Female, 27 years	1.8:1.0
Heskestad et al. (2009) [10]	–	1.7:1.0
Rickels et al. (2010) [27]	–	1.4:1.0
Numminen (2011) [24]	–	1.2:1.0
Katsaragakis et al. (2010) [13]	–	2.6:1.0
Szarpak & Madziala (2011) [37]	42 years	1.9:1.0
	Male, 39 years	
	Female, 47 years	
Pérez et al. (2012) [25]	–	RTA: 2.7:1.0
		Other injury: 1.7:1.0
Stocchetti et al. (2012) [35]	45 years	2.85:1.0
Andelic et al. (2012) [1]	46.7 years	3.35: 1.0.
	Male, 44.8 years	
	Female, 53.2 years	
Mauritz et al. (2014) [21]	44.5 years	1.4:1.0
	Male, 40.9 years; female, 49.9 years	
	Outpatients: male, 35.8 years; female, 38.6 years	
	In-hospital deaths: 65.9 years; male, 62.5 years; female, 73.5 years	
Puljula et al. (2013) [26]	In 1999: overall, 44 years; male, 41 years; female, 52 years. In 2007: overall, 48 years; male, 46 years; female, 55 years	1999: 2.6:1.0
		2007: 3.2:1.0
Scholten et al. (2014) [30]	–	1.35:1.0
Shivaji et al. (2014) [33]	–	2.29:1.0

Mean age varies strongly: Styrke et al. (2007) [36] reported a mean age of 22 years, while Mauritz et al. (2008) [22] reported a mean age of 49 years. The latter study, however, included only severe TBI cases. The variation in mean age probably reflects different case ascertainment and inclusion criteria. In most cases, the mean age in females was higher than the mean age in males.

In all 28 studies, there was a male predominance: the male-to-female ratio ranged from 1.2:1.0 [24] to 4.6:1.0 [22].

In 13 out of 26 studies that provided data on the mechanisms of injury, falls were the most frequent cause of TBI. Road traffic accidents (RTAs) were reported as the most frequent cause of TBI in 11 studies. Table 4 shows the most frequent causes of TBI in the study period and TBI severity. In 8 out of 13 studies that include data from before 2000, RTAs are reported as the main cause of TBI. Falls were dominant in the remaining five studies. Only 2 out of 12 studies that include solely data from 2000 or later report RTA as the main cause of the brain injury. In eight studies, falls were dominant. Thus, over time a clear shift can be seen in terms of leading cause of TBI, namely from RTAs to falls.

**Table 4:** Most frequent cause of TBI in the study period and TBI severity

Reference	TBI severity	Study period	Most frequent cause of TBI
Maegele et al. (2007) [18]	Severe	1990–1999	RTAs (55.3 %)
Koskinen & Alaranta (2008) [14]	All	1991–2005	Falls (51.8 %)
Andersson et al. (2003) [3]	All	1992–1993	Falls (fall from height 27.2 %; fall same level 30.88 %)
Ingebritsen et al. (1998) [12]	All	1993	Falls (62 %)
Firsching & Woischneck (2001) [7]	All	1996	RTAs (56 %)
Servadei et al. (2002) [32]	All	1996	RTAs (48 %)
Masson et al. (2001) [19]	Severe	1996	RTAs (48.3 %)
Masson et al. (2003) [20]	Severe	1996	RTAs (58.9 %)
Stocchetti et al. (2012) [35]	–	1997–2007	–
Baldo et al. (2003) [5]	All	1996–2000	RTAs (49 %)
Servadei et al. (2002) [31]	All	1998	RTA (48 %)
Steudel et al. (2005) [34]	All	1998	–
Shivaji et al. (2014) [33]	All	1998–2009	Falls (47 %)
Puljula et al. (2013) [26]	Moderate-to-severe	1999 & 2007	Falls (1999: 33 %; 2007: 50 %)
Rosso et al. (2007) [29]	Severe	1999–2004	RTA (44 %)
Rickels et al. (2010) [27]	All	2000–2001	Falls (52.5 %)
Pérez et al. (2012) [25]	All	2000–2009	–
Styrke et al. (2007) [36]	All	2001	Falls (55 %)
Mauritz et al. (2008) [22]	Severe	2001–2005	RTAs (41 %)
Numminen (2011) [24]	All	2002–2004	Falls (58.4 %)
Heskestad et al. (2009) [10]	All	2003	Falls (51 %)
Andelic et al. (2008) [2]	All	2005–2006	Falls (51 %)
Andriessen et al. (2011) [4]	Moderate-to-severe	2008–2009	RTA (50 %)
Szarpak & Madziala (2011) [37]	All	2009	Falls (29 %)
Andelic et al. (2012) [1]	Severe	2009–2010	Falls (51 %)
Mauritz et al. (2014) [21]	All	2009–2011	Falls (16.7 % )
Scholten et al. (2014) [30]	All	2010–2012	–
Katsaragakis et al. (2010) [13]	–	–	RTAs (54.1 %)

Within the studies that focus mainly on more severe TBI, RTA as a cause of injury remains dominant. In this category of studies (moderate-to-severe and severe TBI only), RTA remains the leading cause in six out of eight studies.

A clear correlation was also found between age and mechanism of injury. Falls are most common in two age groups: the elderly and children. In contrast, RTAs are the most frequent cause in the age group of young adults. Also notable is the geographical spread of the mechanisms of injury: Scandinavian countries reported mainly falls, while other countries reported more RTAs.

### Mortality rate and case fatality rate

Nine studies reported data on mortality rates (ESM 2). As with the incidence rates, a large variation was found in the mortality rates: from 3.0 per 10^5^ inhabitants per year in Hannover and Münster (Germany) [27] to 18.3 per 10^5^ inhabitants per year in Finland and Romagna (Italy) [14, 31]. This variation can largely be explained by differences in case ascertainment and case definitions. Overall, an average mortality rate of 10.5/100,000 was calculated, but interpretation should be with caution due to the heterogeneity of studies.

The case fatality rate (CFR) expresses disease-specific mortality (e.g. TBI). However, the specificity of the rate is influenced by the inclusion of patients who have died from systemic injuries or non-brain comorbidity. Distinction is made between in-hospital CFR (only in-hospital deaths) and overall CFR (in-hospital and out-of-hospital deaths). CFR is highly dependent on the severity of TBI and age of TBI patients: CFR of TBI in general ranges from 0.9 per 100 patients to 7.6 per 100 patients, while CFR of severe TBI ranges from 29 to 55 per 100 patients. None of the included studies provide information on the difference between CFR in mild TBI compared to severe TBI.

## Discussion

In recent decades, substantial research has been conducted on the epidemiology of TBI in Europe. However, a full profit cannot be taken of this potential because data have not been collected in a uniform way [16]. This review illustrates the great variability, previously reported by Maas et al. (2011) [16], that exists in data collection and coding of variables in TBI studies. Differences in case ascertainment and case definition confound comparisons between and analysis across different studies. A general consensus on choice and coding of variables for TBI studies is needed in order to acquire the exact epidemiological evolution of TBI. This is currently facilitated by the (common data elements, CDEs^1^) In context of the epidemiology, the following categories are of great importance: participant/subject characteristics; participant and family history; injury/disease related events. In general, many reports have focused on participant/subject characteristics, but fewer on the other two categories. The CDEs represent a major advance towards standardisation, which is highly relevant both from a scientific point of view and from the perspective of cost-efficiency, as this will obviate repeated development of case report forms for new studies [16].

Variability in case definitions and case ascertainment does not directly influence the methodological quality of individual studies. However, 9 of the 28 studies included in the review did not meet the quality criteria of the five selected elements of the STROBE checklist. We chose to evaluate the methodological quality of the studies included according to a pre-specified checklist with specific criteria, rather than allocating a subjective judgment. We considered the STROBE checklist [39] as the most appropriate tool, and selected five criteria of this checklist as being most relevant to the evaluation of epidemiological studies. However, despite the use of this checklist and pre-defined criteria, an element of subjective assessment remains. Tables 1 and 3 illustrate the need for improvement of methodological quality, as well as a great need for standardisation of studies and their reporting.

Unlike Tagliaferri et al. [38], who reported an average incidence and mortality rate in their review, we used the random effects model of meta-analysis to calculate an overall incidence rate. This model is better suited for the comparison of studies with a large heterogeneity. Based on the random effects model of meta-analysis, we found an overall incidence rate of 262 per 100,000 per year. For sake of comparison, we also calculated a simple average incidence rate. This average incidence rate (only including the 17 studies focusing on patients with all TBI severities) was about 275 per 100,000 population per year. After excluding the aberrant rates from Spain [25] and Western Sweden [3], the average rate was 326 per 100,000 population per year. This estimate differs greatly from the incidence rate of 235 per 10^5^ population per year reported by Tagliaferri et al. [38] in 2006. This could indicate an increase in incidence of TBI in the past decade or an under-registration of TBI in period 1980–1990. The latter is the most likely explanation in high income countries, while an increase in true incidence of TBI has been described for middle and low-income countries [17].

It remains, however, difficult to calculate an average incidence and mortality rate since great variation can be found in the case definitions, inclusion criteria and methods used in the studies. For example, studies that are based on hospital and emergency department admissions will report a higher incidence rate than studies that are only based on one of these two. For this reason, it is important to interpret the average rates in a critical manner. For morality, we calculated an average rate of 10.53 per 10^5^ per year. This rate is lower than the mortality rate of 15.4 found by Tagliaferri et al. [38] in 2006. Interpretation of this decrease should, however, be viewed with great caution, given the heterogeneity between studies and absence of possibilities to adjust for case mix. Table 5 shows a comparison between the review of Tagliaferri et al. [38] and the current review.

**Table 5:** Comparison with review of Tagliaferri et al. 2006 [38]

	Tagliaferri et al. 2006 [38]	This review
Time period of included studies	1980–2003	1990–2014
Number of included studies	23	28 (9^a^)
Number of countries	12	16
Average incidence rate per 10^5^/year	235	326
Overall incidence rate per 10^5^/year^b^	–	262
Most frequent cause of TBI (number of studies)	RTAs (8) > falls (6)	Falls (14) > RTAs (11)
Sex	Male > female	Male > female
Average mortality rate per 10^5^/year	15	10, 5

^a^Nine studies overlap with the review by Tagliaferri et al. 2006 [38]

^b^Overall incidence derived from random effects modelling

More definitive conclusions can be drawn on changing epidemiological patterns. In most of the studies, a peak is seen in the oldest age groups. Some studies even report an evolution of the mean age over the years. These findings confirm the shift, reported by Roozenbeek et al. (2013) [28], towards older age groups over recent decades, especially in high-income countries. In contrast to Tagliaferri et al. [38], who reported RTA as the most common event leading to TBI, we find falls to be the leading cause. Table 4 clearly shows the shift over time from RTAs to falls as the leading cause of TBI. RTA still remains the most frequent cause in the group of severe TBI. However, an interaction may exist with study period as most of the studies on severe TBI contain data from before 2000.

Falls are thus becoming a more and more important cause of TBI, mainly in the high-income regions of Europe. An additional finding is the strong correlation between age groups and mechanism of injury. In the majority of the studies, we found that falls are more common in the youngest and oldest age group. On the other hand, we found that RTAs are most common in young adults. These differences have important implications for targeting prevention campaigns.

## Strengths and limitations

We used clear search terms and conducted a thorough and systematic literature search. We attempted to include all the relevant articles and to display the study characteristics and results in a clear manner. However, we should note that some studies may have been missed, e.g. if they did not meet the search terms or were not included in PubMed. The major limitations are inherent to the studies underpinning this review and mainly relate to the differences in case ascertainment and case definitions. Although we used the random effects model of meta-analysis to derive an overall incidence rate, the large degree of heterogeneity identified implies that interpretation should be with caution.

## Conclusions

This review does not show any trend towards a decreasing incidence of TBI in Europe. The average mortality rate appears lower than in a previous review. Interpretation of data should, however, be with caution, given existing heterogeneity between reports and major differences in approaches to definitions and case ascertainment. In 2006, Tagliaferri et al. [38] identified a need for high-quality epidemiological studies and collaborative intra-European Union population-based studies. Our review confirms the need for generalised/standardised case definitions, case ascertainment and study methods. We further identify changes in epidemiological patterns with increasing age and identify falls as currently the most common cause of TBI in Europe. This has changed compared with previous studies in which RTAs were the more dominant cause. These changes in epidemiological patterns should inform better targeting of prevention campaigns.

## Electronic supplementary material

ESM 1 (PDF 176 kb)

ESM 2 (PDF 273 kb)

## Footnotes

1 CDEs contain all essential data elements for use across the broad spectrum of TBI. Related elements were combined in modules, which were grouped together in categories. For example, the data elements ‘age, gender and race’ are combined in the module ‘demographics’ under the category ‘subject characteristics’.
