# Supplementary material for: Epidemiology of traumatic brain injury in Europe
Source: Acta Neurochir (Wien). 2015 Aug 14;157(10):1683–96. doi: 10.1007/s00701-015-2512-7 (PMC4569652; doi:10.1007/s00701-015-2512-7)
Supplement: Supplementary file 1 — (PDF 176 kb) [file 701_2015_2512_MOESM1_ESM.pdf]

ESM Table 1. Pubmed search terms

Search terms	Selection criteria	Number of hits
(Brain Injur* [Title/Abstract]) AND (Epidemiology [Title/Abstract])	- English<br>- Full text available<br>- Publication dates: 1990-2014	273
(Head Injur* [Title/Abstract]) AND (Epidemiology [Title/Abstract])	- English<br>- Full text available<br>- Publication dates: 1990-2014	216
(Brain injur*[Title/Abstract]) AND (Incidence[Title/Abstract] AND Europe	- English<br>- Full text available<br>- Publication dates: 1990-2014	125
(Head Injur*[Title/Abstract]) AND Incidence[Title/Abstract] AND Europe	- English<br>- Full text available<br>- Publication dates: 1990-2014	129

Total identified articles	Duplicates	Potentially relevant citations
743	109	634
