# Supplementary material for: Epidemiology of traumatic brain injury in Europe
Source: Acta Neurochir (Wien). 2015 Aug 14;157(10):1683–96. doi: 10.1007/s00701-015-2512-7 (PMC4569652; doi:10.1007/s00701-015-2512-7)
Supplement: Supplementary file 2 — (PDF 273 kb) [file 701_2015_2512_MOESM2_ESM.pdf]

ESM Table 2. TBI mortality rate and case fatality rates

Study location, (reference)	Mortality rate per 10^5 population	In-Hospital No. cases	Case fatality rate		Total case fatality rate	
			No. deaths	per 100[16]	No.deaths	per 100[16]
Aquitaine, France, 1996 (Masson et al, 2001) [19]	5,2	NR	NR	30[1] – 51[2]	NR	NR
Austria, 1999-2004, (Rosso et al, 2007) [29]	NR	NR	NR	31,7[7]	NR	NR
Austria, 2009-2011, (Mauritz et al, 2014) [21]	11	73622	1518	2,1	2665	3,64
Cologne, Germany, 1990-1999 (Maegele et al, 2007) [18]	3,3	468[6]	116	24,8	298	45,8
European regions with different economic status, 2001-2005, (Mauritz et al, 2008) [22]	NR	HI: 406; UMI: 337; LMI: 429[8]	HI: 170; UMI: 162; LMI: 234[8,9]	HI: 42; UMI: 48; LMI: 55[8,9]	NR	NR
Finland, 1991-2005, (Koskinen, 2008) [14]	18,3[10]	27138	1378	5,1[11]	14131	18,1[12]
Germany, 1996 (Firsching, 2001) [7]	11,5	NR	NR	NR	NR	NR
Germany, 2000 (Steudel et al, 2005) [34]	9,4 (1999)	276758 (1999)	2723	1,0	7705 (1999)	2,7
Greece, (Katsaragakis et al, 2010) [13]	NR	3383	144	4,3[6]	389	11,5
Hannover & Münster, Germany, 2000-2001, (Rickels et al, 2010) [27]	3,0[6]	5221	47	0,9[6]	66	0,97[6]
Milan & Monza,Italy, 1997-2007, (Stocchetti et al, 2012) [35]	NR	1366	416[13]	33[13]	NR	NR
Northeast Italy, 1996-2000 (Baldo et al 2003) [5]	7,0[3]	11074[3]	314[4]	3,1[4]	NR	NR
Northern Ostrobothnia, Finland, 1999 and 2007, (Puljula et al, 2013) [26]	NR	NR	NR	NR	156	58[6]
Norway, 2009-2010, (Andelic et al, 2012) [1]	NR	278	80	29	NR	NR
Oslo, Norway, 2005-2006 (Andelic et al, 2008)[2]	NR	445	9	2,0	NR	NR
Romagna, Italy, 1998 (Servadei et al, 2002) [31]	18,3	2890	125	2,8[5]	225	7,8[5]
Romagna & Trentino, Italy, (Servadei et al, 2002) [32]	NR	4442[6]	85[6]	1,9[6]	NR	NR
South East Finland, 2002-2004, (Numminen, 2011) [24]	NR	370	28[14]	7,6[6,14]	NR	NR
The Netherlands, 2008-2009 (Andriessen et al, 2011) [4]	NR	508 (339 severe & 169 moderate TBI)	143	28,1[6]	169 (140 severe & 29 moderate TBI) [15]	33,3 (46 severe & 21 moderate TBI) [6, 15]

[1] overall severe, [2]patients in coma with HAIS of 5, [3] average over 5 years, [4] derived from population average and TBI mortality rate, [5] includes residents, and non-residents , [6] tabled value(s) were calculated from published data, [7] ICU fatality rate, [8] HI: High Income; UMI: Upper Middle Income; LMI: Low Middle Income, [9] after 90 days [10] average over 15 years, [11] in period 2001-2005, [12] in period 1991-2005, [13] after 6 months , [14] in first month, [15] After 6 months follow up, [16] rate/100 patient
